# Prognostic significance of bcl-2 expression in leiomyosarcoma of the uterus

**DOI:** 10.1038/sj.bjc.6690578

**Published:** 1999-07

**Authors:** Y-L Zhai, T Nikaido, T Toki, A Shiozawa, A Orii, S Fujii

**Affiliations:** Department of Obstetrics and Gynecology, Shinshu University School of Medicine, 3-1-1 Asahi, Matsumoto 390-8621, Japan; Department of Gynecology and Obstetrics, Kyoto University Faculty of Medicine, Kyoto 606-8397, Japan

**Keywords:** uterine leiomyosarcoma, bcl-2, p53, prognosis

## Abstract

We examined bcl-2 expression as well as p53 expression and mutation in human uterine smooth muscle tumours to determine the influence of bcl-2 expression on prognosis in patients with uterine leiomyosarcomas. bcl-2 protein was expressed in nearly all benign smooth muscle tumours but in only 57% of leiomyosarcomas. Benign smooth muscle tumours were usually negative for p53 protein, but 16 out of 21 (76%) leiomyosarcomas were positive. A p53 gene mutation was detected in nine of the 16 leiomyosarcomas that showed p53-positive staining. A significant positive correlation was observed between p53 mutation and p53 expression, between the number of mitoses and the Ki-67 labelling index, and between clinical stage and p53 mutation. A significant negative correlation was observed between bcl-2 expression and p53 mutation, and between bcl-2 expression and p53 overexpression. Univariate survival analysis revealed that bcl-2 expression, p53 mutation and clinical stage (stage 1 vs stages 2–4) all showed a significant correlation with prognosis. In a multivariate stepwise regression analysis, positive bcl-2 expression and stage 1 disease were the independent predictors of a favourable prognosis. Our results suggest that bcl-2 is frequently expressed in human uterine smooth muscle tumours, and that its expression may correlate with a favourable prognosis in patients with uterine leiomyosarcoma. © 1999 Cancer Research Campaign


British Journal of Cancer (1999) 80(10), 1658–1664
© 1999 Cancer Research Campaign
Article no. bjoc.1999.0578

### Prognostic significance of bcl-2 expression in leiomyosarcoma of the uterus

**Y-L Zhai[1], T Nikaido[1], T Toki[1], A Shiozawa[1], A Orii[1] and S Fujii[2]**

[1]Department of Obstetrics and Gynecology, Shinshu University School of Medicine, 3-1-1 Asahi, Matsumoto 390-8621, Japan; [2]Department of Gynecology and Obstetrics, Kyoto University Faculty of Medicine, Kyoto 606-8397, Japan

**Summary** We examined bcl-2 expression as well as p53 expression and mutation in human uterine smooth muscle tumours to determine the influence of bcl-2 expression on prognosis in patients with uterine leiomyosarcomas. bcl-2 protein was expressed in nearly all benign smooth muscle tumours but in only 57% of leiomyosarcomas. Benign smooth muscle tumours were usually negative for p53 protein, but 16 out of 21 (76%) leiomyosarcomas were positive. A p53 gene mutation was detected in nine of the 16 leiomyosarcomas that showed p53-positive staining. A significant positive correlation was observed between p53 mutation and p53 expression, between the number of mitoses and the Ki-67 labelling index, and between clinical stage and p53 mutation. A significant negative correlation was observed between bcl-2 expression and p53 mutation, and between bcl-2 expression and p53 overexpression. Univariate survival analysis revealed that bcl-2 expression, p53 mutation and clinical stage (stage 1 vs stages 2–4) all showed a significant correlation with prognosis. In a multivariate stepwise regression analysis, positive bcl-2 expression and stage 1 disease were the independent predictors of a favourable prognosis. Our results suggest that bcl-2 is frequently expressed in human uterine smooth muscle tumours, and that its expression may correlate with a favourable prognosis in patients with uterine leiomyosarcoma.

**Keywords:** uterine leiomyosarcoma; bcl-2; p53; prognosis

Uterine smooth muscle tumours are the most common neoplasm in the female genital tract. They are histologically classified into several subtypes: usual leiomyoma (UL), cellular leiomyoma (CL), bizarre leiomyoma (BL), smooth muscle tumours of uncertain malignant potential (UMP) and leiomyosarcoma (LMS). Benign smooth muscle tumours are common in the uterus, but LMS is a rare tumour, accounting for only about 1.3% of all uterine malignancies (Zaloudek et al, 1994). Uterine LMS usually exhibits an extremely malignant clinical course. The risk of local recurrence and metastasis is high in LMS and the reported 5-year survival rate in cases of uterine LMS is only 12–25% (Zaloudek et al, 1994). The marked difference in prevalence seen between leiomyoma and leiomyosarcoma of the uterus suggests that different mechanisms may underlie the development of these two kinds of smooth muscle tumours.

Alterations in a number of oncogenes and tumour-suppressor genes, resulting in an imbalance between cellular proliferation and programmed cell death (apoptosis), are required for tumour progression (Korsmeyer et al, 1992; Kerr et al, 1994). It has been reported that both overexpression and mutation of tumour-suppressor gene p53 are exhibited by uterine LMSs but not by leiomyomas (de Vos et al, 1994; Jeffers et al, 1995; Zhai et al, 1998), suggesting that a loss of p53 function is implicated in the pathogenesis of LMSs. The wild-type p53 inhibits cell cycling, in cooperation with cyclin-dependent kinase (cdk) inhibitors such as p21 and p27, and induces apoptosis (Yonish-Rouach et al, 1991; Shaw et al, 1992; Hartwell et al, 1994). By contrast, mutant p53 can inhibit apoptosis and stimulate cell division and proliferation (Lotem et al, 1993). Mutation or overexpression of p53 is found increasingly as the stage of the disease advances, implying that p53 alterations occur late in tumour development (Pollock et al, 1996). Moreover, it has been reported that p53 overexpression correlates with a poor prognosis in a number of malignancies (Borg et al, 1995; Lim et al, 1996; Uchiyama et al, 1997).

It is known that bcl-2, as well as p53, is involved in cell cycle regulation and apoptosis (Williams et al, 1991). The *bcl-2* proto-oncogene encodes a 26 kDa protein mainly localized in mitochondrial membranes and this bcl-2 protein has been shown to prolong cell survival by inhibiting the apoptotic processes induced by many anticancer drugs, radiation and DNA-damaging agents, processes that are mediated by p53 (Hockenbery et al, 1990; Sentman et al, 1991; Korsmeyer et al, 1992; Miyashita et al, 1993; Wang et al, 1993). bcl-2 expression has been demonstrated in a variety of human cancers (Colombel et al, 1993; Joensuu et al, 1994) and it has been reported that bcl-2 expression is of prognostic significance in a number of malignancies (Baretton et al, 1996; Nakanishi et al, 1997; Nakopoulou et al, 1998), as is the expression of p53. In some cancers, bcl-2 expression correlates with negative p53 staining (which implies the presence of wild-type p53) (Silvestrini et al, 1994; Alderson et al, 1995; Fontanini et al, 1995). Mutant p53 can substitute functionally for bcl-2 and can down-regulate bcl-2 expression (Haldar et al, 1994), suggesting that bcl-2 expression should be more common in tumours containing wild-type p53 (Silvestrini et al, 1994; Alderson et al, 1995). Thus, the relationship between bcl-2 and p53 in malignant tumours is of interest, but very few studies have involved simultaneous investigation of both bcl-2 and p53 expression in uterine LMSs. In this study, we investigated the expression of bcl-2 and p53, as well as of p53 mutation, in uterine smooth

*Received 12 October 1998*
*Revised 12 January 1999*
*Accepted 27 January 1999*

*Correspondence to:* T Nikaido muscle tumours and we assessed the prognostic significance of these molecules in cases of LMS.

### MATERIALS AND METHODS

### Tissue collection

Sixty-seven patients (32–70 years of age) diagnosed as having smooth muscle tumours of the uterus were selected from the pathology file of Shinshu University Hospital. The tissues were used after obtaining written consent from the patients or their next of kin. Tissue blocks, proteins and DNA were prepared from the removed uteri. The fresh tissues were fixed in 10% phosphate-buffered formalin and embedded in paraffin. Serial sections were prepared for haematoxylin and eosin staining, immunohistochemistry and p53-mutation analysis. The pathological diagnosis of uterine smooth muscle tumours was performed using the accepted criteria (Hendrickson et al, 1995). Mitotic activity was evaluated by counting the number of mitoses in ten consecutive high-power fields and it was expressed as a mitotic index (MI). In this study, the diameter of one high-power field (HPF) was approximately 0.65 mm. Of the 67 cases of smooth muscle tumours, 21 were diagnosed as LMS, eight were smooth muscle tumours of UMP, eight were BL, 15 were CL and 15 were UL. From these cases, fresh uterine tissues (from ten cases of normal myometrium, ten cases of UL and three cases of LMS) were used for Western blotting, and paraffin sections of 21 LMSs and 25 leiomyomas (five ULs, four CLs, eight UMPs and eight BLs) were used for DNA sequencing. The clinical stage of LMS was determined according to the FIGO classification of endometrial carcinomas (Creasman et al, 1989). Clinical information, including follow-up data, was obtained from the medical records.

### Immunohistochemistry

Immunohistochemical analysis for bcl-2, p53 and Ki-67 was performed on serial paraffin sections of the 67 smooth muscle tumours. In cases of LMS, we employed at least two different tissue blocks from each case. The p53 staining and Ki-67 labelling index data for all the leiomyomas and 14 out of the 21 leiomyosarcomas have already been reported by us (Zhai et al, 1998), and those data were included in our assessment of the correlation between p53 and bcl-2 expression in the present study. The primary antibodies used were bcl-2 (Dako, Copenhagen, Denmark), p53 (DO-1; Immunotech, Marseille, France) and Ki-67 (MIB-1; Immunotech). The avidin–biotin–peroxidase complex method was performed using a Histofine SAB-PO (M) detector kit (Nichirei, Tokyo, Japan). In brief, the sections were deparaffinized in xylene, rehydrated through graded alcohols and treated in a 0.01 M citrate buffer (pH 6.0) for 15 min in a microwave oven. They were then incubated with 0.03% hydrogen peroxide to block endogenous peroxidase activity, and with normal rabbit serum to reduce non-specific binding. The sections were incubated with a specific primary antibody or non-immunized mouse serum at 4°C overnight. Biotinylated rabbit anti-mouse IgG was used as a linker. The sections were incubated with the streptavidin–biotin complex and stained with diaminobenzidine. The sections were lightly counterstained with haematoxylin.

### Interpretation of immunohistochemical staining

The staining of p53 and bcl-2 was interpreted independently by three observers (YZ, TT and SF). The results, based on the percentage of stained cells among 1000 arbitrarily selected tumour cells, were expressed as follows: ++, diffusely positive (more than 50% of the tumour cells were stained); +, partially positive (5–49% of the cells were stained); and –, negative (less than 5% of the cells were stained). The percentage of Ki-67-positive cells among 1000 arbitrarily selected tumour cells was also counted and the result was expressed in the form of a Ki-67 labelling index.

### Western blot analysis

To confirm the antigenic specificity of the antibody used and also to obtain quantitative information, the expression of bcl-2 protein was examined by Western blotting. Fresh uterine tissues (normal myometrium, ten cases; usual leiomyoma, ten cases; leiomyosarcoma, three cases) were homogenized using a Polytron (Kinematica, Switzerland), then lysed in 1 ml of cell-lysis buffer. The buffer contained 50 mM Tris–HCl (pH 8.0), 0.25 M sodium chloride, 0.5% NP-40, 1 mM phenylmethyl sulphoxide (Sigma), 1 mg $ml^{-1}$ aprotinin (Boehringer Mannheim, Mannheim, Germany), 1 mg $ml^{-1}$ leupeptin (Boehringer Mannheim) and 20 mg $ml^{-1}$ TPCK (Boehringer Mannheim). Lysates were centrifuged at 13 000 ***g*** for 20 min at 4°C and the supernatants were stored at –80°C. Extracts equivalent to 50 μg of total protein were separated by sodium dodecyl sulphate polyacrylamide gel electrophoresis (12% acrylamide gel), followed by the equilibration of the gel in transfer buffer (20% methanol, 25 mM Tris, 192 mM glycine, pH 8.0) for 30 min. The proteins were then transferred to supported nitrocellulose membranes (Gibco BRL, Gaithersburg, MD, USA) by applying 2400 V-min using a plate-electrode apparatus (Idea Scientific, Minneapolis, MN, USA). Filters were blocked for 1 h in TBST (0.2 M sodium chloride, 10 mM Tris, pH 7.4, 0.2% Tween-20) containing 5% non-fat dry milk and 0.02% sodium azide. This was followed by incubations with monoclonal antibodies for bcl-2 (1:500 dilution; Dako) and β-actin (1:2000 dilution; AC-15, Biomakor, Rehovot, Israel, as the internal standard) in TBST containing 5% non-fat milk overnight at 4°C, then with anti-mouse IgG (1:1000; Amersham, Arlington Heights, IL, USA) in TBST containing 2% non-fat milk. The filters were washed several times with TBST between steps. Bound antibody was detected by means of an enhanced chemiluminescence (ECL) system (Amersham, Aylesbury, UK) and exposed to X-ray films.

### Polymerase chain reaction-DNA sequencing of p53

To help us determine whether overexpression of p53 is caused by a mutation of the p53 gene, we examined the sequences of the DNA from all the 21 LMSs and 25 of the leiomyomas (including those with positive p53 staining) using the DyeDeoxy method. The sequence analysis of the p53 gene in 14 out of the 21 leiomyosarcomas and in the same 25 leiomyomas has already been reported by us (Zhai et al, 1998). In the present study, we studied p53 mutation in seven more cases of leiomyosarcoma, and we are reporting the results of all the cases together in this paper. DNAs were extracted from paraffin-embedded consecutive tissue sections using the microwave-based DNA extraction method for polymerase chain reaction (PCR) amplification (Banerjee et al, 1995). Exons 5–8 were amplified by PCR according to the published sequencing of oligonucleotide primers, as described previously (Oda et al, 1992). After heating at 94°C for 2 min, all samples of genomic DNA underwent thermocycling for 35 cycles (94°C for

© 1999 Cancer Research Campaign

**Table 1** Results of immunohistochemistry for bcl-2 and p53 in smooth muscle tumours of the uterus

| History | bcl-2 | p53 |
|---|---|---|
| MY (*n* = 15) | –/+ | – |
| UL (*n* = 15) | +/++ | – |
| CL (*n* = 15) | +/++ | – |
| BL (*n* = 8) | +/++ | –/+ |
| UMP (*n* = 8) | +/++ | –/+ |
| LMS (*n* = 21) | –/++ | –/++ |

MY, myometrium; UL, usual leiomyoma; CL, cellular leiomyoma; BL, bizarre leiomyoma; UMP, tumours of uncertain malignant potential; LMS, leiomyosarcoma.

1 min, 58°C or 60°C for 1 min, 72°C for 2 min) using a Gene *Taq* Kit (Nippon Gene, Toyama, Japan). To confirm the success of the PCR reaction for each DNA sample, 5 µl of PCR product were electrophoresed on a 2% agarose gel, then each product was purified using a DNA recollectional filter tube (TaKaRa suprec-TM$^{02}$; Takara, Japan). The nucleotide sequences were determined by the dideoxynucleotide-chain-termination method using a Tag Dyedeoxy Terminator Cycle Sequencing Kit (Applied Biosystems, Foster City, CA, USA).

### Statistical analysis

The Kruskal–Wallis rank test and Scheffé's F test were used to examine the significance of differences in the Ki-67 labelling index among the smooth muscle tumours. Spearman's rank correlation was used to determine whether there was a significant positive or negative correlation between any two of the results. Cox's proportional hazard model was used to identify the significant predictors of survival (SPSS v7.5; SPSS Inc., Chicago, IL, USA). The prognostic factors used in the survival analysis were the following: the age of the patient (< 50 vs ≥ 50), clinical stage (1 vs 2,3 and 4), mitotic index (< 50 vs ≥ 50), bcl-2 expression (< 5% vs ≥ 5%), p53 overexpression (< 5% vs ≥ 5%), p53 mutation (positive vs negative) and the Ki-67 labelling index (< 25% vs ≥ 25%). Since all the six cases whose disease was at stage 3 or 4 had died of their disease, the comparison was performed between stage 1 and stages 2–4. The cut-off values for the mitotic index and the Ki-67 labelling index were chosen so that the *P*-value became minimal in the univariate analysis. The univariate analysis was first performed on each of the factors. Overall survival was then analysed by means of the step-wise regression model (forward) using those variables with a *P*-value of < 0.4 in the univariate analysis. A *P*-value less than 0.05 was considered significant.

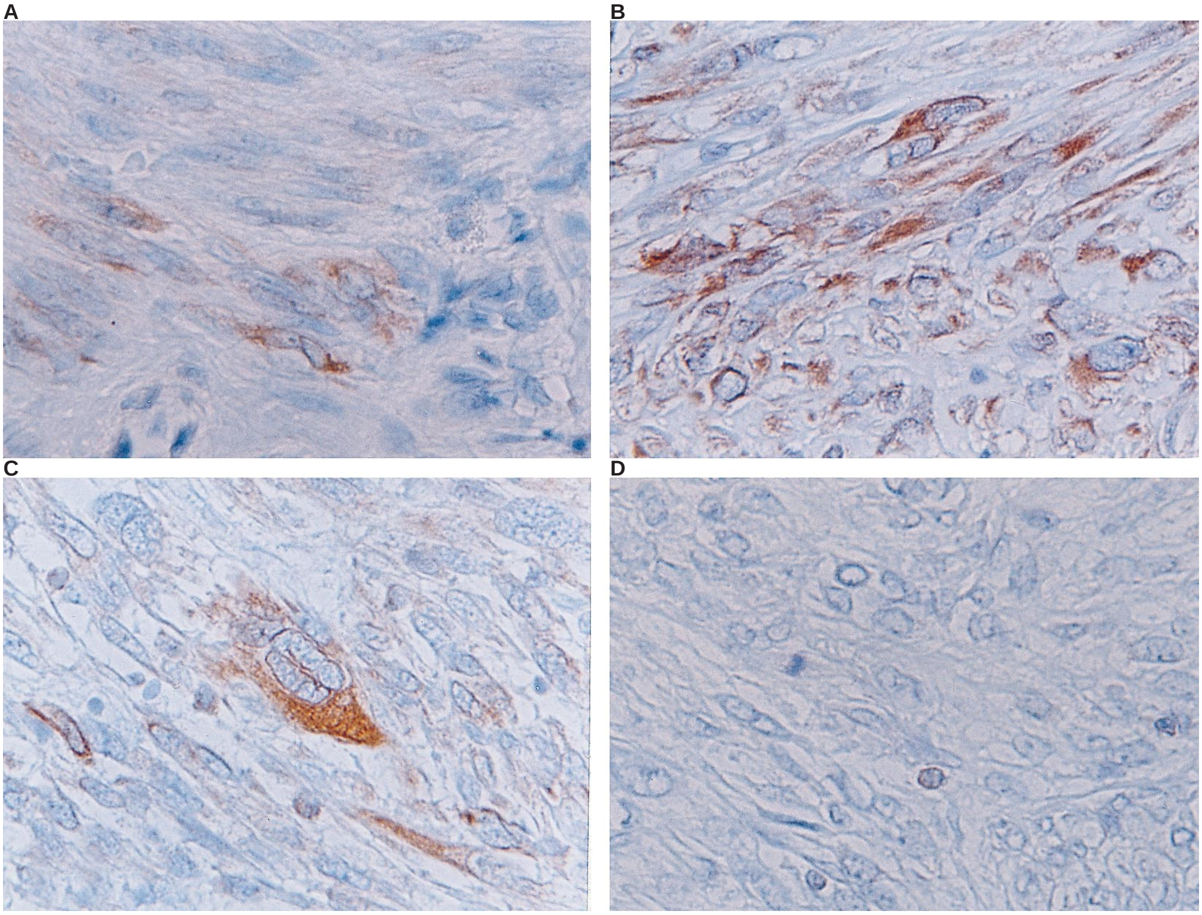

**Figure 1** Immunohistochemical staining for bcl-2. (**A**) Normal myometrium showing focal positive staining for bcl-2, (**B**) leiomyoma showing diffuse positive staining for bcl-2, (**C**) a case of leiomyosarcoma showing positive staining for bcl-2 and (**D**) another case of leiomyosarcoma showing negative staining for bcl-2 (original magnification × 400)

 *© 1999 Cancer Research Campaign*

**Table 2** Clinical profile, Ki-67, bcl-2 and p53 expression, p53 mutation and survival in cases with uterine leiomyosarcoma

| Case | Age | Stage | MI | Ki-67 (%) | bcl-2 | p53 stain | p53 mutation | | Survival (months) |
|---|---|---|---|---|---|---|---|---|---|
| | | | | | | | Codon | Base change (Amino acid) | |
| 1[a] | 37 | 4 | 97 | 25 | – | ++ | 249 | AGG to TGG (Arg to Trp) | D (1) |
| 2 | 69 | 4 | 87 | 52 | – | ++ | 276 | GCC to GAC (Ala to Asp) | D (8) |
| 3 | 70 | 4 | 133 | 43 | – | ++ | 205 | TAT to TGT (Tyr to Cys) | D (5) |
| 4 | 39 | 3 | 85 | 45 | – | ++ | 273 | CGT to CAT (Arg to His) | D (8) |
| 5[a] | 65 | 1 | 30 | 23 | – | ++ | 303 | AGC to ATC (Ser to Ile) | D (20) |
| 6 | 56 | 1 | 53 | 41 | – | ++ | 173 | GTG to ATG (Val to Met) | A (6) |
| 7 | 51 | 1 | 43 | 25 | – | ++ | 215 | AGT to AGG (Ser to Arg) | D (2) |
| 8[a] | 49 | 1 | 46 | 48 | – | ++ | | ND | D (24) |
| 9[a] | 52 | 1 | 107 | 37 | – | + | | ND | D (13) |
| 10[a] | 45 | 2 | 32 | 33 | + | ++ | 285 | GAG to GAC (Glu to Asp) | D (24) |
| 11[a] | 58 | 3 | 24 | 32 | + | + | 248 | CGG to CAG (Arg to Gln) | D (23) |
| 12[a] | 43 | 3 | 57 | 22 | + | – | | ND | D (10) |
| 13[a] | 55 | 1 | 75 | 46 | + | – | | ND | D (18) |
| 14[a] | 67 | 1 | 23 | 21 | + | – | | ND | A (41) |
| 15 | 43 | 2 | 87 | 51 | + | – | | ND | A (14) |
| 16[a] | 51 | 1 | 93 | 38 | + | – | | ND | A (101) |
| 17[a] | 67 | 1 | 64 | 29 | + | + | | ND | A (22) |
| 18[a] | 67 | 1 | 37 | 19 | + | + | | ND | A (23) |
| 19[a] | 51 | 1 | 22 | 13 | ++ | + | | ND | A (41) |
| 20 | 63 | 2 | 43 | 31 | ++ | + | | ND | A (17) |
| 21[a] | 48 | 1 | 14 | 24 | ++ | ++ | | ND | A (66) |

MI, mitotic index; Ki-67, Ki-67 labelling index; D, died of disease; A, alive with no evidence of disease; ND, not detected. [a]Ki-67 labelling index, p53 staining and *p53* gene mutations in these cases have been reported by our previous study (Zhai et al, 1998).

### RESULTS

### Immunohistochemistry in uterine smooth muscle tumours

Specific staining was identified as a brown colour in the nuclei for p53 and Ki-67 and in the cytoplasm for bcl-2. All the control slides yielded negative staining. The results of the immunohistochemistry are summarized in Table 1 for the various uterine smooth muscle tumours. Normal myometrial tissue adjacent to the benign smooth muscle tumours was focally positive for bcl-2 (Figure 1A). All the cases of UL, CL, BL and UMP were diffusely positive for bcl-2 (Figure 1B), except for three cases in which bcl-2 expression was equivocal, and the staining intensity was generally stronger in the leiomyomas than in the myometria. Of the 21 cases of LMS, nine (43%) were partially positive and three (14%) were diffusely positive for bcl-2 (Figure 1C), while nine (43%) were negative (Figure 1D). Lymphocytes scattered in the smooth muscle tumours were often positive for bcl-2 as well. UL, CL, most UMP, most BL and the adjacent myometrium were negative for p53 (Figure 2A) but one (out of nine) UMP and two (out of nine) BLs were partially positive for p53. Of the 21 LMSs, 16 (76%) were partially or diffusely positive for p53 (Figure 2B) and five were negative (Table 2). The Ki-67 labelling index was significantly higher in LMS (32.0 ± 12.0; mean ± s.d.) than in UL (0.5 ± 0.3), CL (0.6 ± 0.5), BL (0.5 ± 0.6) or UMP (1.5 ± 1.0) ($P < 0.001$).

### Western blotting of bcl-2

Tissue samples from myometrium or leiomyoma showed a specific band for bcl-2 with a molecular weight of 26 kDa (Figure 3, lanes 1–6). Among the samples from leiomyosarcomas, two (Figure 3, lanes 7 and 8; cases 17 and 18 in Table 2) showed weak bands and one (Figure 3, lanes 9; case 20 in Table 2) showed a strong specific band for bcl-2 (Figure 3). These results were compatible with those obtained by immunostaining.

### Detection of p53 mutation

No mutation of the p53 gene was detected in any of the 25 leiomyomas (including the one UMP and two BLs that showed positive staining for p53). In LMS, a mis-sense mutation of the p53 gene was detected in nine specimens. Of these nine, one showed a mutation in exon 5, two a mutation in exon 6 and in exon 7, and four a mutation in exon 8. All of these nine cases were diffusely positive for p53 by immunostaining. However, no mutation was detected in the remaining 13 LMSs, irrespective of their p53 immunoreactivity (Table 2).

### Relationships among clinical stage, mitotic index, immunohistochemical results and p53 mutation in leiomyosarcoma

Table 3 summarizes the correlations between pairs of the following factors in cases of LMS: clinical stage, mitotic index, bcl-2 expression, p53 expression, p53 mutation and the Ki-67 labelling index. Significant positive correlations were observed between p53 expression and p53 mutation (correlation coefficient 0.65, $P < 0.005$), between stage and p53 mutation (correlation coefficient 0.59, $P < 0.005$) and between mitotic activity and the Ki-67 labelling index (correlation coefficient 0.47, $P < 0.05$). On the other hand, significant negative correlations were observed between bcl-2 expression and p53 mutation (correlation coefficient –0.62, $P < 0.005$) and between bcl-2 expression and p53 expression (correlation coefficient –0.45, $P < 0.05$).

### Survival analysis

Among the 21 patients with LMS, 12 had died of their disease and nine were alive with no evidence of disease. The mean

© 1999 Cancer Research Campaign

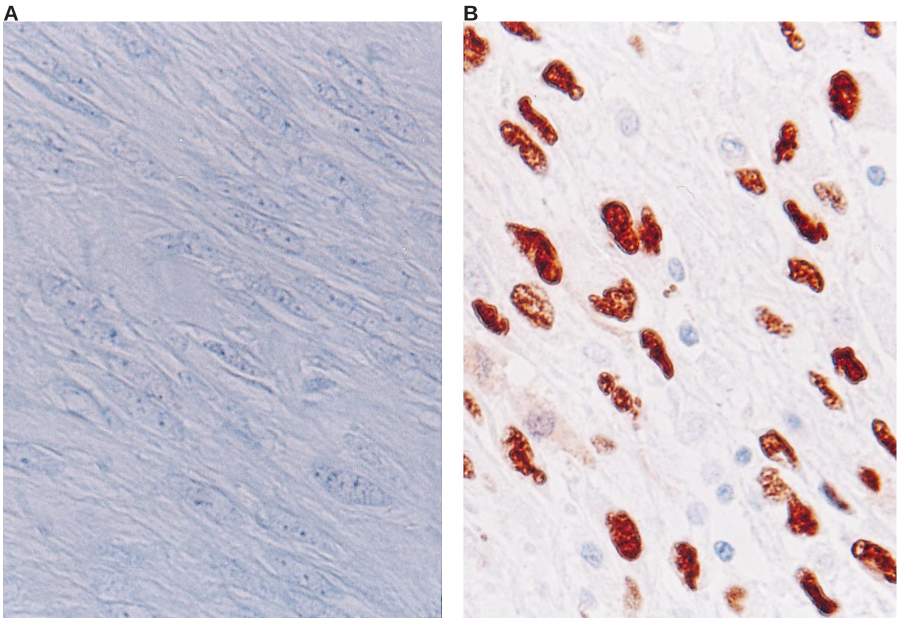

**Figure 2** Immunohistochemical staining for p53. (**A**) Usual leiomyoma showing negative staining for p53, (**B**) leiomyosarcoma showing diffuse positive staining for p53 (original magnification × 400)

**Table 3** Correlations between the clinicopathological parameters examined in uterine leiomyosarcoma

| | Mitotic index | bcl-2 expression | p53 expression | p53 mutation | Ki-67 index |
|---|---|---|---|---|---|
| Clinical stage | 0.33 | –0.28 | 0.3 | 0.59[b] | 0.31 |
| Mitotic index | | –0.33 | 0.1 | –0.03 | 0.47[a] |
| bcl-2 expression | | | –0.45[a] | –0.62[b] | –0.42 |
| p53 expression | | | | 0.65[b] | 0.093 |
| p53 mutation | | | | | 0.17 |

Data are presented as correlation coefficients; [a] $P < 0.05$; [b] $P < 0.005$.

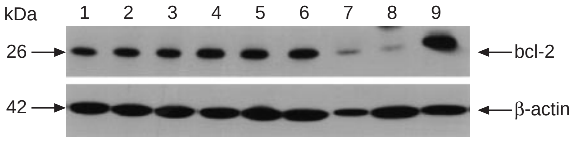

**Figure 3** Results of Western blotting for bcl-2 and β-actin. Lanes 1–3: myometrium, lanes 4–6: leiomyoma, lanes 7–9: leiomyosarcomas. Myometrium and leiomyoma are showing specific bands for bcl-2 (molecular weight 26 kDa). The cases in lanes 7 and 8 (weak bands) correspond to cases 17 and 18 in Table 2, and the case in lane 9 (distinct band) corresponds to case 20 in Table 2

**Table 4** Univariate and stepwise regression analyses of the prognostic value of clinical and histopathological variables in 21 patients with uterine leiomyosarcoma

| | Disease-free survival | | | |
|---|---|---|---|---|
| **Prognostic factor** | **Univariate (*P*)** | **RR (CI)** | **Stepwise (*P*)** | **RR (CI)** |
| Age <50 | 0.64 | 1.3 (0.4–4.2) | not included | |
| Stages 2, 3 and 4 | 0.041[a] | 3.4 (1.1–11.3) | 0.0083[b] | 9.3 (1.8–48.9) |
| Mitotic index >50 | 0.082 | 3.0 (0.9–10.0) | 0.086 | |
| Negative bcl-2 | 0.0028[c] | 6.6 (1.9–22.5) | 0.0012[c] | 15.4 (3.0–80.6) |
| Positive p53 staining | 0.39 | 1.9 (0.4–8.9) | >0.4 | |
| Positive p53 mutation | 0.0062[b] | 5.5 (1.6–18.7) | >0.4 | |
| Ki-67 labelling index > 25 | 0.079 | 3.9 (0.9–18.0) | >0.2 | |

RR, relative risk; CI, 95% confidence intervals; [a] $P < 0.05$; [b] $P < 0.01$; [c] $P < 0.005$.

post-operative observation period was 34.7 ± 30.3 months. A univariate analysis using Cox's proportional hazard model revealed that the prognosis was significantly poorer in patients (i) with negative bcl-2 expression ($P < 0.005$), (ii) with a p53 mutation ($P < 0.01$) and (iii) nine cases at stages 2, 3 or 4 as compared to 12 cases at stage 1 ($P < 0.05$) (Table 4). The age of the patients ($P = 0.64$) was not included in the step-wise regression. The step-wise regression analysis (forward) was performed using six variables (clinical stage, mitotic index, bcl-2 expression, p53 staining, p53 mutation status and the Ki-67 labelling index).

 *© 1999 Cancer Research Campaign*

A longer disease-free survival was significantly correlated with positive bcl-2 expression ($P = 0.0012$) and stage 1 disease ($P = 0.0083$, as compared to stages 2–4) (Table 4).

### DISCUSSION

This study has revealed the presence of a stronger bcl-2 expression in benign uterine smooth muscle cell tumours (UL, CL, BL and UMP) than in the normal myometrium or in leiomyosarcomas. The Western blotting experiments supported this evidence of an over-production of bcl-2 protein in these benign tumours. The benign leiomyomas showed negative or weak positive staining for p53, no p53 mutation and a low Ki-67 labelling index. The bcl-2 overexpression in benign uterine smooth muscle tumours suggests that it might play an important role in preventing apoptosis among benign neoplastic smooth muscle cells. Although bcl-2 itself does not stimulate cell growth, an inhibition of apoptosis by bcl-2 may provide a survival advantage to the cells of benign uterine smooth muscle tumours. However, since cellular proliferation and apoptosis form a complex mechanism, another pathway, not involving bcl-2 and p53, could also be implicated in the regulation of the cell cycle and cell growth in benign leiomyomas of the uterus.

In the leiomyosarcomas, we noted a strong positive correlation between p53 expression and p53 mutation. While wild-type p53 was generally correlated with weak or negative p53 staining. However, in a few cases, there was an apparent discrepancy between p53-mutation status and p53 staining. In fact, half of the tumours that were positive for p53 did not show a mutation in exons 5–8 of the p53 gene examined, but it is possible that abnormalities of the p53 gene were present in other exons. To judge from these results, although the overall correlation between p53 mutation and p53 expression is very good, p53 staining may not be completely consistent with p53-mutation status.

In this study, a strong negative correlation was found between bcl-2 expression and p53 mutation, and a less significant one was found between bcl-2 expression and p53 expression. Previously, an inverse correlation between bcl-2 and p53 expression has been reported in a number of human malignancies (Pezzella et al, 1993; Silvestrini et al, 1994; Alderson et al, 1995; Fontanini et al, 1995; Kaklamanis et al, 1998). Most of these studies were performed using immunohistochemical methods alone and only one investigated p53 mutation simultaneously (Alderson et al, 1995, Lepelley et al, 1995). The results of the present study suggest that p53 mutation, rather than p53-positive staining or immunoreactivity, is inversely correlated with bcl-2 expression. Interestingly, a fairly recent study indicated that mutant p53 can down-regulate bcl-2 expression in breast cancer cells (Haldar et al, 1994). These results, taken together, suggest that the reduced expression of bcl-2 seen in some leiomyosarcomas might well have been induced by mutant p53. However, wild-type p53 induces a decrease in bcl-2 expression in vitro (Miyashita et al, 1994), while in vivo studies on human malignant tumours (including the present study) have shown that weak p53 expression, which indicates that p53 is not mutated but of the wild type, is correlated with an increased bcl-2 expression (Pezzella et al, 1993; Silvestrini et al, 1994; Alderson et al, 1995). Since diffuse cell loss by apoptosis eventually leads to an arrest of tumour growth, a regulation of apoptosis would be an appropriate target for genetic changes associated with malignant tissue transformation. Our results suggest that an inhibition of the induction of apoptosis – either by bcl-2 overexpression (in most of the benign leiomyomas and almost a half of the leiomyosarcomas) or by p53 inactivation due to its mutation (in the remaining half of the leiomyosarcomas) – might be a common phenomenon in human uterine smooth muscle tumours.

It has been reported that clinical stage (Wolfson et al, 1994; Nola et al, 1996), tumour size (Evans et al, 1988), mitotic activity (Major et al, 1993), DNA index (Wolfson et al, 1994) and p53 overexpression (Niemann et al, 1995) correlate with prognosis in uterine leiomyosarcomas. In the present study, a univariate survival analysis showed that survival was significantly correlated with bcl-2 expression, p53 mutation and clinical stage. A subsequent step-wise regression analysis revealed that bcl-2 expression and clinical stage 1 disease (as compared to stages 2–4) were the best independent predictors of a favourable prognosis. In fact, the prognostic significance of bcl-2 expression has already been reported in a number of malignancies. Most reported a favourable prognosis in patients with tumours positive for bcl-2 (Pezzella et al, 1994; Silvestrini et al, 1994; Fontanini et al, 1995; Kaklamanis et al, 1998), although bcl-2 expression correlated with a poor prognosis in another report (Brambilla et al, 1996). Thus, the prognostic significance of bcl-2 may vary among different types of malignant tumours. Although the biological significance of bcl-2 expression in malignant tumours is not entirely clear, bcl-2 expression does appear to play an important role in the growth of human carcinomas. In particular, the diffuse bcl-2 expression in benign uterine smooth muscle tumours and the good clinical outcome of LMSs with a bcl-2 expression together suggest that bcl-2 might possibly be involved in the inhibition of tumour progression or spread.

In conclusion, the present study has shown that bcl-2 is frequently expressed in human uterine smooth muscle tumours. A significant positive correlation between clinical stage and p53 mutation status and a significant negative correlation between bcl-2 expression and p53-mutation status was observed among uterine leiomyosarcomas. A multivariate survival analysis revealed that bcl-2 expression and stage 1 disease (as compared to stages 2–4) were significantly correlated with a favourable prognosis in patients with uterine leiomyosarcoma.

### ACKNOWLEDGEMENTS

This work was supported in part by Grants-in-aid for Scientific Research from the Ministry of Education, Science, Sports and Culture (No. 08457438, 09877318, 09671671, 09877318, 10470345), Japan.

### REFERENCES

Alderson LM, Castleberg RL, Harsh GR IV, Louis DN and Henson JW (1995) Human gliomas with wild-type p53 express bcl-2. *Cancer Res* **55**: 999–1001

Banerjee SK, Makdisi WF, Weston AP, Mitchell SM and Campbell DR (1995) Microwave-based DNA extraction from paraffin-embedded tissue for PCR amplification. *BioTechniques* **18**: 768–770, 772–773

Baretton GB, Diebold J, Christoforis G, Vogt M, Müller C, Dopfer K, Schneiderbanger K, Schmidt M and Lohrs U (1996) Apoptosis and immunohistochemical bcl-2 expression in colorectal adenomas and carcinomas. *Cancer* **77**: 255–264

Borg A, Lennerstrand J, Stenmark-Askmalm M, Ferno M, Brisfors A, Ohrvik A, Stal O, Killander D, Lane D and Brundell J (1995) Prognostic significance of p53 overexpression in primary breast cancer; a novel luminometric immunoassay applicable on steroid receptor cytosols. *Br J Cancer* **71**: 1013–1017

Brambilla E, Negoescu A, Gazzeri S, Lantuejoul S, Moro D, Brambilla C and Coll J-L (1996) Apoptosis-related factors p53, Bcl-2, and Bax in neuroendocrine lung tumors. *Am J Pathol* **149**: 1941–1952

Colombel M, Symmans F, Gil S, O'Toole KM, Chopin D, Benson M, Olsson CA, Korsmeyer S and Buttyan R (1993) Detection of apoptosis-suppressing oncoprotein bcl-2 in hormone-refractory human prostate cancers. *Am J Pathol* **143**: 390–400

Creasman WT (1989) Announcements, FIGO stages: 1988 revision. *Gynecol Oncol* **35**: 125–126

de Vos S, Wilczynski SP, Fleischhacker M and Koeffler P (1994) p53 alterations in uterine leiomyosarcomas versus leiomyomas. *Gynecol Oncol* **54**: 205–208

Evans HL, Chawla SP, Simpson C and Finn KP (1988) Smooth muscle neoplasms of the uterus other than ordinary leiomyoma. *Cancer* **62**: 2239–2247

Fontanini G, Vignati S, Bigini D, Mussi A, Lucchi M, Angeletti CA, Basolo F and Bevilacqua G (1995) Bcl-2 protein: a prognostic factor inversely correlated to p53 in non-small-cell lung cancer. *Br J Cancer* **71**: 1003–1007

Haldar S, Negrini M, Monne M, Sabbioni S and Croce CM (1994) Down-regulation of bcl-2 by p53 in breast cancer cells. *Cancer Res* **54**: 2095–2097

Hartwell LH and Kastan MB (1994) Cell cycle control and cancer. *Science* **266**: 1821–1828

Hendrickson MR and Kempson RL (1995) Pure mesenchymal neoplasms of the uterine corpus. In: *Obstetrical and Gynecological Pathology*, Fox H and Wells M (eds), pp. 542–573. Churchill Livingstone: Edinburgh

Hockenbery D, Nunez G, Milliman C, Schreiber RD and Korsmeyer SJ (1990) Bcl-2 is an inner mitochondrial membrane protein that blocks programmed cell death. *Nature* **348**: 334–336

Jeffers MD, Farquharson MA, Richmond JA and McNil AM (1995) p53 immunoreactivity and mutation of the p53 gene in smooth muscle tumours of the uterine corpus. *J Pathol* **177**: 65–70

Joensuu H, Pylkkanen L and Toikkanen S (1994) Bcl-2 protein expression and long-term survival in breast cancer. *Am J Pathol* **145**: 1191–1198

Kaklamanis L, Savage A, Whitehouse R, Doussis-Anagnostopoulou I, Biddolph S, Tsiotos P, Mortensen N, Gatter KC and Harris AL (1998) Bcl-2 protein expression: association with p53 and prognosis in colorectal cancer. *Br J Cancer* **77**: 1864–1869

Kerr JF, Winterford CM and Harmon BV (1994) Apoptosis. Its significance in cancer and cancer therapy. *Cancer* **73**: 2013–2026

Korsmeyer SJ (1992) Bcl-2 initiates a new category of oncogenes: regulators of cell death. *Blood* **80**: 879–886

Lepelley P, Soenen V, Preudhomme C, Merlat A, Cosson A and Fenaux P (1995) bcl-2 expression in myelodysplastic syndromes and its correlation with hematological features, p53 mutations and prognosis. *Leukemia* **9**: 726–730

Lim BH, Soong R, Grieu F, Robbins PD, House AK and Iacopetta BJ (1996) p53 accumulation and mutation are prognostic indicators of poor survival in human gastric carcinoma. *Int J Cancer* **69**: 200–204

Lotem J and Sachs L (1993) Regulation by bcl-2, c-myc, and p53 of susceptibility to induction of apoptosis by heat shock and cancer chemotherapy compounds in differentiation-competent and -defective myeloid leukemic cells. *Cell Growth Differ* **4**: 41–47

Major FJ, Blessing JA, Silverberg SG, Morrow CP, Creasman WT, Currie JL, Yordan E and Brady MF (1993) Prognostic factors in early-stage uterine sarcoma. *Cancer* **71**: 1702–1709

Miyashita T and Reed JC (1993) Bcl-2 oncoprotein blocks chemotherapy-induced apoptosis in a human leukemia cell line. *Blood* **81**: 151–157

Miyashita T, Krajewski S, Krajewska M, Wang HG, Lin HK, Liebermann DA, Hoffman B and Reed JC (1994) Tumor suppressor *p53* is a regulator of *bcl-2* and *bax* gene expression in vitro and in vivo. *Oncogene* **9**: 1799–1805

Nakanishi H, Ohsawa M, Naka N, Uchida A, Ochi T and Aozaka K (1997) Immunohistochemical detection of bcl-2 and p53 protein and apoptosis in soft tissue sarcoma: their correlations with prognosis. *Oncology* **54**: 238–244

Nakopoulou L, Vourlakou C, Zervas A, Tzonou A, Gakiopoulou H and Dimopoulos MA (1998) The prevalence of bcl-2, p53, and Ki-67 immunoreactivity in transitional cell bladder carcinomas and their clinicopathologic correlates. *Hum Pathol* **29**: 146–154

Nola M, Babic D, Llic J, Marusic M, Uzarevic B, Petrovecki M, Sabioncello A, Kovac D and Jukic S (1996) Prognostic parameters for survival of patients with malignant mesenchymal tumors of the uterus. *Cancer* **78**: 2543–2550

Oda T, Tsuda H, Scarpa A, Sakamoto M and Hirohashi S (1992) Mutation pattern of the p53 gene as a diagnostic marker for multiple hepatocellular carcinoma. *Cancer Res* **52**: 3674–3678

Pezzella F, Turley H, Kuzu I, Tungekar MF, Dunnill MS, Pierce CB, Harris A, Gatter KC and Mason DY (1993) bcl-2 protein in non-small-cell lung carcinoma. *N Engl J Med* **329**: 690–694

Pollock RE, Lang A, Luo J, El-Naggar AK and Yu D (1996) Soft tissue sarcoma metastasis from clonal expansion of p53 mutated tumor cells. *Oncogene* **12**: 2035–2039

Sentman CL, Shutter JR, Hockenbery D, Kanagawa O and Korsmeyer SJ (1991) bcl-2 inhibits multiple forms of apoptosis but not negative selections in thymocytes. *Cell* **67**: 879–888

Shaw P, Bovey R, Tardy S, Sahli R, Sordat B and Costa J (1992) Induction of apoptosis by wild-type p53 in a human colon tumor-derived cell line. *Proc Natl Acad Sci USA* **89**: 4495–4499

Silvestrini R. Veneroni S, Daidone MG, Benini E, Boracchi P, Mezzetti M, Di Fronzo G, Rilke F and Veronesi U (1994) The bcl-2 protein: a prognostic indicator strongly related to p53 protein in lymph node-negative breast cancer patients.
*J Natl Cancer Inst* **86**: 499–504

Uchiyama M, Iwasaka T, Matsuo N, Hachisuga T, Mori M and Sugimori H (1997) Correlation between human papillomavirus positivity and p53 gene overexpression in adenocarcinoma of the uterine cervix. *Gynecol Oncol* **65**: 23–29

Wang Y, Szekely L, Okan I, Klein G and Wiman KG (1993) Wild-type p53-triggered apoptosis is inhibited by bcl-2 in a v-myc-induced T-cell lymphoma line. *Oncogene* **8**: 3427–3431

Williams GT (1991) Programmed cell death: apoptosis and oncogenesis. *Cell* **65**: 1097–1098

Wolfson AH, Wolfson DJ, Sittler SY, Breton L, Markoe AM, Schwade JG, Houdek PV, Averette HE, Sevin BU, Penalver M, Duncan RC and Ganjei P (1994) A multivariate analysis of clinicopathologic factors for predicting outcome in uterine sarcomas. *Gynecol Oncol* **52**: 56–62

Yonish-Rouach E, Resnitzki D, Lotem J, Sachs L, Kimchi A and Oren M (1991) Wild-type p53 induces apoptosis of myeloid leukaemic cells that is inhibited by interleukin-6. *Nature* **352**: 345–347

Zaloudek CJ and Norris HJ (1994) Mesenchymal tumors of the uterus. In: *Blausteins's Pathology of the Female Genital Tract*, Kurman RJ (ed), pp. 849–914. Springer-Verlag: New York

Zhai YL, Kobayashi Y, Mori A, Orii A, Nikaido T, Konishi I and Fujii S (1999) Expression of steroid receptors, Ki-67, and p53 in uterine leiomyosarcomas. *Int J Gynecol Pathol* **18**: 20–28

*© 1999 Cancer Research Campaign*
